# Relevance of a Novel Circuit-Level Model of Episodic Memories to Alzheimer’s Disease

**DOI:** 10.3390/ijms23010462

**Published:** 2021-12-31

**Authors:** Krisztián A. Kovács

**Affiliations:** Retina Research Laboratory, Institute of Translational Medicine, Semmelweis University, Tűzoltó U. 37-47, 1094 Budapest, Hungary; kovacs.krisztian@med.semmelweis-univ.hu or kkovacs.ist@t-online.hu; Tel.: +36-20-315-9746

**Keywords:** Alzheimer’s disease, episodic memories, entorhinal cortex, hippocampus, reelin, EPISODE module, neurogenesis, synaptogenesis, granule cells, anterograde amnesia

## Abstract

The medial temporal lobe memory system has long been identified as the brain region showing the first histopathological changes in early Alzheimer’s disease (AD), and the functional decline observed in patients also points to a loss of function in this brain area. Nonetheless, the exact identity of the neurons and networks that undergo deterioration has not been determined so far. A recent study has identified the entorhinal and hippocampal neural circuits responsible for encoding new episodic memories. Using this novel model we describe the elements of the episodic memory network that are especially vulnerable in early AD. We provide a hypothesis of how reduced reelin signaling within such a network can promote AD-related changes. Establishing novel associations and creating a temporal structure for new episodic memories are both affected in AD. Here, we furnish a reasonable explanation for both of these previous observations.

## 1. Introduction

Alzheimer’s disease (AD) is the most common cause of dementia in older adults and therefore the burden it constitutes will increase in the developed countries with the foreseeable demographic changes. The cognitive decline in AD is progressive with distinct functions of the central nervous system becoming affected at different stages of the disease and this has been suggested to correlate with the spreading of pathological phenomena from the transentorhinal cortex along particular anatomical pathways to other regions of the brain [1]. One of the earliest cognitive deficits includes anterograde episodic memory impairment followed by retrograde episodic memory impairments intruding more and more profoundly into the past [2]. We have recently developed a circuit-level theory describing the encoding of new episodic memories in the entorhino–hippocampal system based on a particular entorhinal neuronal ensemble that we termed EPISODE module [3]. This concept provides a new molecular, cellular, and network-level explanation of what clinical observations show in patients with early phases of AD. Namely, we attribute the earliest subtle deficits of anterograde episodic memories, both verbal and non-verbal [2], to the compromised synaptogenesis between the axons originating from the EPISODE modules and the newborn granule cells of the dentate gyrus. Our model also accounts well for the decreased delayed temporal contiguity that accompanies the earliest phases of cognitive decline [4]. We hypothesize that this deterioration is linked to an uncoupling between the phase code generated by the EPISODE modules and ensuing deficits of sequence generation in the hippocampal CA3 area. Finally, a remarkable further link between the EPISODE modules and the pathomechanism of AD is reelin, a large secreted protein. On the one hand, reelin signaling is proven to change in AD, and research on how rescuing reelin signaling could remedy patients with AD became more and more intensive in the last decade. On the other hand, a key subset of superficial entorhinal cortical cells building up the EPISODE modules is one of the very few excitatory neuronal populations that express reelin in the adult central nervous system [5]. By describing a novel mechanism of early AD based on specific molecular, cellular, and network-level changes, we aim to facilitate translational AD research in three different ways: (i) defining where the reelin signaling needs to be restored within the central nervous system; (ii) finding new molecular targets; and (iii) increasing the efficiency of psychometric tests used for early diagnosis.

## 2. Results

### 2.1. The Reelin-Positive Excitatory Entorhinal Neurons Constitute a Link between Our Recent Circuit-Level Model of Episodic Memories and the Pathomechanism of AD

The stellate and fan cells in the pre-α layer of the entorhinal and transentorhinal cortex are unique as reelin-expressing excitatory neurons since the presence of reelin is normally restricted to inhibitory interneurons in the human adult central nervous system with the notable exception of the mitral cells of the olfactory bulb [6] and the glutamatergic cerebellar granule cells [7]. Reelin is a large extracellular signaling molecule orchestrating the layering of the cerebral cortex during development, the differentiation of the neurons migrating during the ontogenesis, and the maturation of the dendritic spines [8]. Of key importance, in adulthood, reelin is supposed to carry a similar role in the fascia dentata of the hippocampus by providing guidance cues to the newborn granule cells. Presumably, it also acts as a stop signal for these neurons similarly to its role during the development of the central nervous system [8]. Reelin exerts its effect at the neurophysiological level as well: it can facilitate the learning-induced plasticity at particular synapses in rodents as evidenced by measuring the long-term potentiation [9]. Experiments in adult animals have demonstrated that blocking the intra-entorhinal reelin signaling triggered a spatial learning deficit that prevented rodents from remembering particular positions in the watermaze [10].

Recently reelin came into the focus of research on AD and mild cognitive impairment (MCI). The entorhinal and transentorhinal regions of the human brain that harbors the reelin positive stellate and fan cells are the first to show alterations that can be detected right from the onset of MCI by assessing the thickness of the entorhinal and transentorhinal cortex [11]. In patients with very mild AD, these are the regions where the first histopathological changes can be observed, moreover, it is the population of layer II neurons (equivalent to the layer pre-α) that is subjected to a profound loss of cells [12]. In line with this finding, the entorhinal pre-α is the first region of the human brain where neurofibrillary tangles and neuropil threads can be detected [13], and the reelin-positive cells of this layer selectively express intracellular amyloid already in the mildest form of AD [14].

Taken together, the entorhinal reelin positive excitatory neurons are supposed to be exceptionally fragile to AD-related changes that might spread from these cells later to other areas of the brain [15]. Therefore it is reasonable to assume that the early decrease of connectivity without histopathological and structural alterations described in the human anterior temporal system by Berron et al. [16] is mainly happening within the EPISODE modules that we have previously defined [3]. Thus, we did a comprehensive research to extract data from the available literature on the relationship between reelin, the pathomechanism of AD, and adult hippocampal neurogenesis that is required by our model for novel associations (see Section 2.2). The key results are presented in Table 1, and strongly support our core hypothesis that AD is initiated by reelin hypofunction within the EPISODE modules. Of note, the increase of reelin expression in the frontal cortex, hippocampus, and cerebrospinal fluid of AD patients contrasts the decrease in the entorhinal cortex and is typically considered a compensatory change (Table 1).

**Figure 1:**
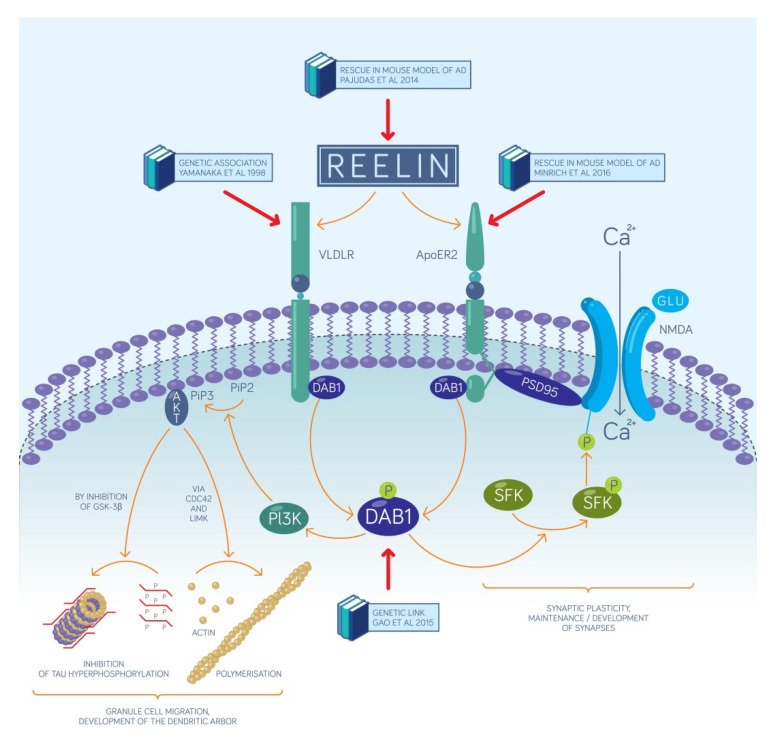
The key actors of reelin signaling. The two neuronal receptors of reelin are depicted in green, along with their downstream effector molecules. On the left, we show the mechanism of how the cellular migration and the dendritogeneis are promoted by reelin, while on the right the main intracellular pathway leading to synaptic plasticity and synaptogenesis is highlighted. Both mechanisms are essential for the newborn granule cells to establish functional contacts with entorhinal input axons. More than two decades of research link AD to a deficit of adult reelin signaling hence some key reports describing prominent links to particular signaling molecules are indicated and coupled to those molecules with a red arrow.

Reelin and the molecules in its signaling pathway hold a great promise to become the main targets for developing drug molecules against AD. Reelin depletion is evident in the entorhinal cortex of amyloid precursor protein mutant mice and AD patients, furthermore, this depletion correlates well with the severity of the disease [19]. Overexpression of reelin was shown to be efficient to counteract the decline in an established mouse model of AD, including the rescue of the performance in the novel object recognition test [28]. The latest molecular mechanism published explains this effect of reelin by hypothesizing that in the pre-plaque stage, soluble forms of Aβ first inhibit reelin signaling which in turn decreases GSK3β inhibition leading to the hyperphosphorylation of the τ-protein [34]. This results in deficits of the microtubule-based intracellular transport in the entorhinal cortex [34] (Figure 1). This mechanism is supported by reports showing that mice either lacking reelin or both of its receptors show an increased level of τ-phosphorylation [35,36]. It is therefore reasonable to assume that in AD, the seed region of τ-hyperphosphorylation lies within the entorhinal EPISODE module, and this molecular alteration spreads to other brain areas via anatomical connections, as it has previously been described [1]. We summarize the most important molecules of the reelin signaling pathways in Figure 1 and the most important observations that link them to AD. These molecular mechanisms elucidate how the cellular mechanisms of episodic memory formation, published in our latest model [3], become compromised in patients with AD due to reelin depletion.

Several potential molecular targets of future drug development against AD are depicted in Figure 1 and any intervention that increases the amplitude of reelin signaling without substantial adverse effects is assumed to be beneficial. Of particular interest, ApoE4, the molecule that has the strongest genetic link with AD, has been shown to interact with one of the two reelin receptors, ApoER2, and reduces its cell surface expression by inhibiting its recycling [37]. Consequently, all drug candidates that disrupt the interaction between ApoE4 and ApoER2 hold a great promise as therapeutic agents. From the point of view of target-based drug development, the clinical importance of our new network model lies in specifying the precise location within the brain where reelin signaling needs to be restored. Recognizing the key role of the EPISODE modules in AD implies that reelin signaling initiated by the excitatory entorhinal neurons has to be reinstated specifically rather than reelin signaling in general or that mediated by reelin-positive inhibitory interneurons. Genetic tools may soon become available to achieve such specificity, and, importantly, at least some target neurons are preserved in early AD although their reelin expression drops [19]. A promising genetic tool for restoring reelin expression is the Sim1 promoter that allowed for the selective targeting of fan cells and stellate cells of the mouse superficial entorhinal cortex [38], although it remains to be elucidated whether similar selectivity can be achieved in the human brain. The Sim1 or promoters with similar activity profiles could make up the foundation of vector-based gene therapy for AD. Finally, as detailed in Section 2.2 below, the creation of new episodic memories requires the granule neurons of the fascia dentata to be responsive to reelin signaling that they receive from the upstream entorhinal EPISODE modules. Therefore, any intervention that selectively increases Dab1 expression or activity in these cells could potentially be turned into a therapeutic approach against AD.

### 2.2. Synaptogenesis Described by Our Circuit-Level Model Underlie the Encoding of New Associations that Become Deficient Early on in AD

Prodromal AD, a more sharply defined condition than MCI, is characterized by episodic memory deficits that can be revealed using psychiatric tests of cued and free recall after an appropriate delay [39]. These deficits can be almost as severe in prodromal AD as they are in demented AD patients, and out of all memory processes, the consolidation stage is reported to be affected [39]. Experiments on animals have shown that one of the key processes in the hippocampus underlying episodic memory consolidation is the activity of granule neurons of the dentate gyrus [40]. These cells constitute one of the two unique populations that can recruit new neurons generated in adulthood. In animals, adult neurogenesis in this area is required for contextual and spatial memory [41], moreover, also for the maintenance of hippocampal memory capacity [42]. In humans with AD, adult hippocampal neurogenesis is drastically reduced hinting at the paucity of new granule neurons as a key reason behind impaired episodic memory [43].

**Figure 2:**
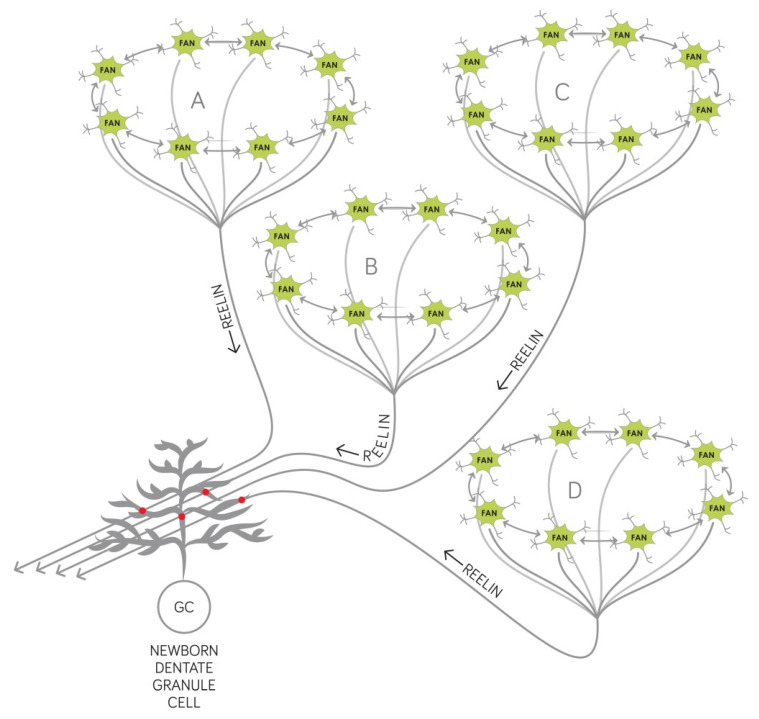
Molecular, cellular, and network mechanisms of encoding new associations. The entities to be linked together in an episodic memory trace have previously encoded representations in the central nervous system that are relayed to the hippocampus by the ring attractors located in the EPISODE modules of the entorhinal cortex. Namely, entities A, B, C, and D are respectively represented by their own A, B, C, and D ring attractors which are activated by relevant sensory input, integrated input derived from multimodal external information, or internally generated input arriving actually to the entorhinal cortex. Axons from neurons (depicted in green, labeled as “FAN” cells) constituting the ring attractors reach the hippocampus via the perforant pathway. When encoding new episodic memories, new associations require newborn dentate granule cells (depicted as “GC”) that are of the right receptive age to form new synapses (indicated as red dots) with such axons. Reelin (see Figure 1) is indispensable for the newborn cells to become postsynaptic cells in this circuit and is provided by the output cells (fan cells or stellate cells) of the EPISODE module. New associations are stored at the level of the granule cells since these neurons establish contacts with a unique set (in the depicted example: A, B, C, and D) of EPISODE modules. For details, the reader is referred to Reference [3].

We have recently developed a model based on adult hippocampal neurogenesis and defined the entorhinal EPISODE module consisting of superficial neurons of the entorhinal cortex as the structure which is responsible for encoding new episodic memories [3]. Each active EPISODE module is supposed to code for an already learned entity that can be of variable complexity and is to be incorporated into a new episodic memory trace [3] (Figure 2). The entorhinal cortex receives highly integrated information from a wide range of cortical areas and this pattern of connectivity supports the EPISODE modules to establish a 1-dimensional code for any entity, regardless of its complexity [3]. All entorhinal EPISODE modules that are active when a new event is encountered are assumed to establish synapses with the newborn dentate granule cells that have the appropriate receptive age at the occurrence of the event (Figure 2). We had hypothesized that this is the principal mechanism that ensures the non-overlapping representation of memory episodes since each new dentate granule cell stores an association with a new unique set of existing EPISODE modules [3]. This concept is in line with the fact that hippocampal neurogenesis adds new neurons to surviving old ones during the whole lifespan of a mouse and this process results in measurable tissue growth [44].

It is reasonable to assume that reelin is a key factor promoting the creation and reinforcement of new synapses between the axons stemming from the EPISODE modules and the dendrites of the receptive newborn granule cells. Reports have shown that in rats, reelin produced in the entorhinal stellate and fan cells is transported down along their axons and released from the terminals in the dentate gyrus [6]. There are several ways that reelin secreted this way can facilitate the synaptogenesis, the most important and best documented of these are the followings:

(1) Reelin affects the proliferation in the subgranular zone where new dentate granule cells are generated. This has been demonstrated in the Reeler mouse that has no reelin expression in any of its tissues [45].
(2) Reelin controls the migration of newborn dentate granule cells within the mouse dentate gyrus. It acts as an attractive signal pulling the young neurons through the already established granule cell layer and guiding them toward the marginal zone of the dentate gyrus [46].
(3) Reelin signaling has an effect on the types and the morphology of the dendritic spines located on the dendritic tree of mouse dentate granule neurons [33] and cell-autonomous inactivation of the reelin receptor Dab1 specifically in developing mouse granule cells impairs their dendritic development [32].

In a middle-aged human, approximately 700 newborn granule neurons are added each day to the fascia dentata [47] and animal studies indicate that these cells fully integrate into the existing circuitry, receive input from the layer II cells of the lateral entorhinal cortex and send output to the CA3 region of the cornu ammonis [48,49].

Therefore, we postulate that as a result of reelin depletion in prodromal AD (see Section 2.1), the birth and migration of the newborn granule cells become impaired as well as the creation of their new synaptic contacts that enable them to receive input from the entorhinal cortex. Consequently, memory traces of new associations are not created which leads to the well-described deficits of anterograde episodic memory [50]. In line with this concept, the perforant pathway furnishing information from layer II (pre-α) of the entorhinal cortex to the fascia dentata is shown to be destroyed in early AD [51].

Recently, it has been questioned whether hippocampal neurogenesis persists into adulthood in humans [52], and a couple of studies failed to detect the assumed young neurons using immunohistochemical methods on post-mortem adult human tissue [53]. Others find the amount of evidence in favor of the adult human hippocampal neurogenesis convincing [54], and such evidence even includes the detection of neural progenitor cells in the live human brain [55]. Importantly, our hypothesis remains valid even if new neural progenitors are not generated in the adult hippocampus. It is still reasonable to assume that prolonged maturation, postmitotic migration, and positioning, furthermore integration into the entorhino–hippocampal circuit and synaptogenesis is reelin-dependent.

Importantly, our recent model [3] also provides a powerful theoretical framework to choose the appropriate cognitive tests to be used in clinical examinations aimed at distinguishing prodromal AD from other forms of cognitive impairment related to normal aging. Furthermore, cognitive tests already used in clinical practice can be redesigned and improved based on the new concept, as detailed below.

When testing the recall of defined sets of words, objects, or sentences, a delayed task is to be opted for, given that the compromised associations rely on the consolidation of new synapses between the lateral entorhinal cortex and the fascia dentata. Furthermore, cued recall is also to be tested since the association of the cue to the other learned items is also assumed to be impaired in the entorhino–hippocampal circuitry. Both the cue and the rest of the items are supposed to be represented by their own EPISODE module and none of them can become connected to the newborn granule cells if synaptogenesis is impaired. Finally, semantic interference should be a very good indicator of prodromal AD, as already evidenced by the excellent psychometric power of the LASSI-L test [56,57]. Our model provides a rationale for this observation: in the absence of successfully reinforced new associations, the subset of active granule neurons is expected to be defined by information encoded before the learning, and such information can already be at least partially semanticized. However, we hypothesize that a slightly modified version of the LASSI-L test where the different sets of words belonging to the same category are presented on different days should be an even stronger predictor of prodromal AD. We propose that recall in the modified LASSI-L be tested after a period sufficiently long for consolidation, this way the lack or the decrease of new synapses between the EPISODE modules activated by the event and the newborn granule cells should be even more conspicuous.

### 2.3. Our Circuit-Level Model Explains Why the Temporal Structure of Episodic Memories Deteriorate at the Onset of AD

**Figure 3:**
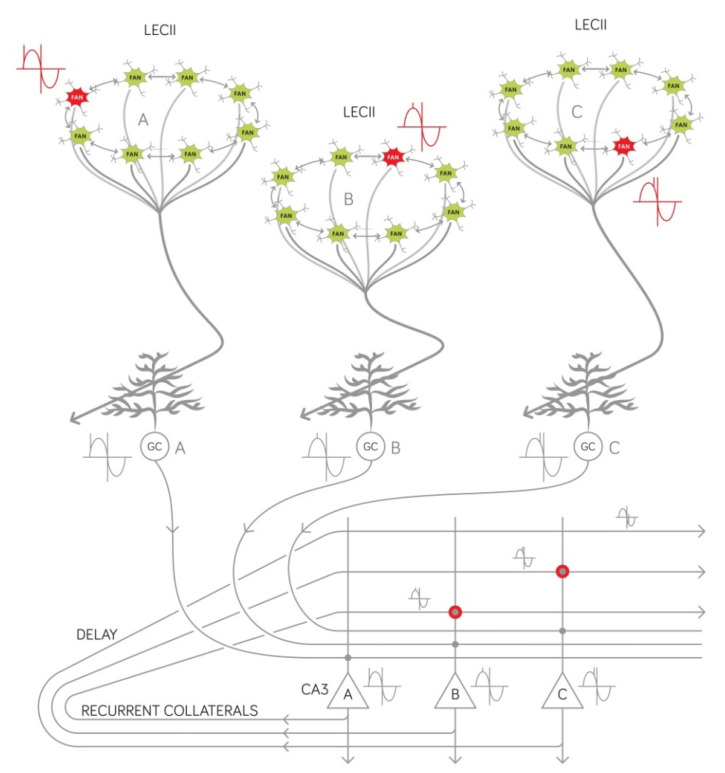
Molecular, cellular, and network mechanisms of encoding sequences. The internal temporal structure of any episodic memory is established by first assigning a theta phase (red inset diagram depicted next to the ring attractors) to each entity represented (three are shown in the Figure: A, B, and C) by a currently active EPISODE module. This theta phase serves to indicate whether the entity is upcoming, current, or already experienced and is repeated several times as several theta cycles unfold during the mnemonic episode to be encoded. As the temporal status of an entity (A, B, or C) changes, a directly measurable phenomenon, the so-called “phase precession” [59] is observed. Importantly, the generation and the repetition of the phase code in the entorhinal cortex is simply explained by the intrinsic electrophysiological and network properties of ring attractors. The theta phase is first transmitted to the dentate granule cells (depicted as “GC”) via the perforant pathway, then to the pyramidal CA3 neurons of the hippocampus via the mossy fibers as indicated by inset diagrams. Sequences are generated in the recurrent network of the CA3 using the phase code derived from the entorhinal EPISODE modules by the following mechanism: adjacent elements of the sequence produce coincident signals at the recurrent CA3-CA3 synapses given that the earlier signal (such as “A” in the Figure) suffers a delay when traveling along the recurrent collaterals and thus becomes coincident with a later one (such as “B” in the Figure) thereby making NMDA dependent plasticity possible (grey dot encircled with red). Sequences are generated by linking adjacent pairs first (in the Figure binding B to A and binding C to B is shown). The Figure is provided only to demonstrate the principle, therefore, several aspects are strongly simplified: (i) further cell types of the entorhinal ring attractors are not shown; (ii) the theta phase is also shifted during the entorhino–hippocampal transmission of the signal (inheritance of the phase precession), however, this results in the same amount of delay for all the entities (A, B, C) thus this aspect is not shown, and (iii) all the entities are represented by a population of cells, therefore, a population of “A” neurons becomes bound to a population of “B” neurons in CA3, etc.; however, for the sake of simplicity, only one neuron represents each population in the Figure. For details, the reader is referred to Reference [3].

One salient feature of our recently published model is that it describes at the circuit level how the inherent temporal structure of new episodic memories emerges [3]. Briefly, within each entorhinal EPISODE module, a theta phase is assigned to each entity that will become part of the episodic memory (Figure 3). The entity can be of any complexity that previous learning and stored semantic information permits while the theta phase represents, on the subsecond timescale, whether the entity (i) is expected, (ii) is present or unfolding, or (iii) belongs to the past. The theta phase of a given entity can change as the event being encoded unfolds [3]. The output of each EPISODE module is sent to the dentate gyrus by the superficially located reelin-positive entorhinal neurons. As described in Section 2.2. above, new associations can be created when different, actually active EPISODE modules establish permanent new synaptic contacts with newborn granule cells that have the right receptive age. Importantly, from the point of view of sequence generation, the granule cells of the fascia dentata, including the older ones, are supposed to simply transmit the theta phase of the entity encoded (Figure 3), as underscored by animal studies reporting the spreading the entorhinal theta phase precession into different hippocampal subregions [58]. We have previously proposed that entities having subsequent theta phases are finally linked together into a meaningful memory sequence in the CA3 region of the hippocampus [3]. Specifically, the representations of two entities juxtaposed along the theta wave are linked together to form a pair by reinforcing specific synapses of the recurrent network in the CA3 (Figure 3). Longer sequences are built the same way by further linking the posterior item of a pair to another representation that was activated even later [3]. Therefore, the reelin positive neurons not only create associations by synaptizing onto the newborn granule cells but also define the order within a memory sequence by assigning a theta phase to each item that will become part of an episodic memory trace. Given that these cells are the first to show pathological alterations in prodromal AD (as described in Section 2.1), it can be expected that the temporal structure of episodic memories will also be compromised early on as AD develops. For the meaningful entorhinal theta phase and thus the hippocampal sequences to emerge, the reelin-positive neurons have to be connected according to a particular topology within the entorhinal EPISODE module. At present, it is not clear whether the entorhinal intralayer synapses of these cells are the first to deteriorate in AD or those that these cells establish with downstream target neurons in the dentate gyrus. Elucidating this will necessitate further histopathological studies, while, when designing psychometric tests, the possibility should be left open that AD affects sequence memory even before the first signs of an anterograde episodic memory deficit.

The molecular, cellular, and network mechanism of generating sequences in the CA3 that we have previously proposed (Figure 3) can also be exploited to improve the psychometric AD tests currently used in the clinical practice given that this mechanism uses the precessing theta phase code [59] provided by the reelin-positive neurons of the entorhinal EPISODE module. Clinicians have long been aware of the fact that the intrinsic sequential nature of an episodic memory trace develops spontaneously as evidenced by the fact that subjects tend to recall items in a similar order as they were presented during the learning phase. This is referred to as temporal contiguity [4]. In line with our theoretical considerations, the delayed temporal contiguity is a good predictor of slipping from the status of cognitively unimpaired, stable (CUS) to the status of cognitively unimpaired, declining (CUD) [4]. Of note, the CUD was previously termed early mild MCI; thus, it is a condition that could precede prodromal AD, although CUD is an umbrella term that includes further conditions preceding other forms of dementia as well.

Therefore, based on the circuit mechanisms that we propose, appropriate tests of temporal contiguity could be used as an early clinical diagnostic tool to follow the development of prodromal AD and the changes associated with the later stages of the disease. Moreover, we propose that highly quantitative approaches to measure the temporal structures of episodic memories [60] should become the gold standard to reliably detect prodromal AD. As already established, healthy subjects can remember the position of a particular frame from a 28-min-long episode of a television show with an average error of 153.3 s [60]. This capacity provides a sufficiently large dynamic range to measure any decline that can accompany the development of AD. Of note, the precision of identifying the temporal positions of the frames correlates well with the fMRI signal in the lateral entorhinal cortex [60] that harbors the reelin-positive excitatory neurons first attacked by AD. However, a simple assessment of the temporal structure of the memory could be more feasible and cost-effective, while fMRI may still be used as a confirmatory measurement.

In the auditory verbal learning test (AVLT), often used to reinforce the diagnosis of AD, subjects tend to best remember the last word during immediate recall. This phenomenon is termed the recency effect [61]. However, when delayed performance is tested, individuals with AD tend to show the most pronounced deficit at remembering the last word. Based on this observation it has been proposed that the ratio between the recency effect at the immediate and at the delayed recall, termed recency ratio (Rr), could be a reliable measure of the decline on the way to AD [61]. This experimental observation is again in full accordance with our network model of episodic memory [3]. At immediate recall, it is the last word that is kept most efficiently in working memory, however, at delayed recall, the last word stands at the last position of a sequence the elements of which activate each other in the CA3 region of the hippocampus (Figure 3). In line with these considerations, recalling sequences of line drawings could also uncover MCI in an earlier study [62]. Specifically, subjects had to reconstruct sequences of 3, 4, or 5 line drawings immediately after they were presented or after a delay of 10 min. The immediate recall could only differentiate between the normal age effect and MCI when the largest span length (five items) was used. On the contrary, delayed recall readily differentiated normal aging from MCI, regardless of span length [62].

Based on this observation we propose that more efficient psychometric tests can be constructed by increasing the delay both in AVLT and in line drawing-based temporal order memory assessments. Ideally, if the experimental paradigm permits, recall should be tested on the subsequent day. Additionally, sequence-based tests with larger span lengths should be validated by following a group of CUD and CUS subjects over a longer period and correlating the evolution of test scores with the development of prodromal AD. We predict that psychometric tests using a longer delay and larger span length will be a very reliable and sensitive predictor of AD.

## 3. Discussion

With decades of clinical investigation, it became unequivocal that AD can be sharply distinguished from other forms of dementia. Early histopathological studies have uncovered characteristic molecular and cellular changes in the brain and defined typical routes of spreading between different brain structures. More recently, several reports hinted at the causative role of decreased reelin signaling in AD without clarifying how such molecular change could interfere with the function of the medial temporal lobe memory system. Here, we have presented a novel concept of how reelin hypofunction and decreased hippocampal neurogenesis can lead to an increasingly strong anterograde declarative memory deficit in AD. The new hypothesis also accounts for the so-far unexplained observation that the temporal structure of new episodic memories is compromised from the earliest moment of disease appearance. To unravel the role of reelin in the early decline of cognitive function, we relied on our recent model that explains at the single neuron, oscillation, network, and electrophysiological level (Figure 2 and Figure 3) how new episodic memories are encoded [3]. These new theoretical considerations will significantly benefit the patients with AD, on the one hand by helping the development of more efficient diagnostic tests, and on the other hand by identifying new molecular targets, and specifying in which neurons exactly the function of these molecules have to be impacted to fight the disease.

## 4. Methods

Literature searches were carried out in PubMed, Scopus, and Web of Science; they were based on the title, keyword, and abstract, and covered a period of 30 years (1991–2021). For hypothesis construction, Boolean operators were used and the following principal terms: “reelin”, “Alzheimer’s disease”, and “adult neurogenesis”. Each possible pair of the principal terms were coupled with the “AND” operator. Potential synonyms were taken into account by building up each principal term using the “OR” operator from multiple terms which were the following: (i) for reelin: “reelin”, “RELN”, “ETL7”, “LIS2”, “PRO1598”, and “RL; (ii) for Alzheimer’s disease: “Alzheimer’s disease”, “Alzheimer’s”, “dementia”, “mild cognitive impairment”, “MCI”, and “tauopathy”; (iii) for adult neurogenesis: “adult neurogenesis”, “adult hippocampal neurogenesis”, “AHN”, “newborn granule cells”, “neurogenesis”, “hippocampal neurogenesis”, “neural progenitor cells”, and “adult neuronal progenitor cells”. Prospective searches in Web of Science were used to find all the reports citing a given study of particular importance. These searches did not reveal any publication that would contradict our core hypothesis. The most important reports supporting the hypothesis are listed in Table 1 and are cited within the text.

## 5. Conclusions

The physiology, the intrinsic molecular signaling pathways, and the network architecture of the neurons composing the entorhinal EPISODE module that we have recently defined [3] can sufficiently explain all major features of the cognitive decline in early AD if considered jointly with the process of adult hippocampal neurogenesis. Future research is expected to develop new diagnostic and therapeutic processes based on this novel concept.

## Figures and Tables

**Table 1:** Summary of relevant previous research on the relationship between reelin, AD, and hippocampal neurogenesis.

Type of Relationship	Year of Publication	Molecule Involved	Finding
Change of reelin expression in AD patients	2003	protein	increase in the cerebrospinal fluid [17]
	2006	protein, mRNA	increase in the frontal cortex [18]
	2007	protein	decrease in the entorhinal cortex [19]
	2010	protein	increase in the frontal cortex [20]
	2012	protein, mRNA	decrease in the entorhinal cortex [21]
	2016	protein	increase in the frontal cortex and the hippocampus [22]
	2020	mRNA	increase in the frontal cortex [23]
Established genetic links with molecules of the reelin signaling pathway in human subjects with AD	1998	VLDLR	[24]
	2008	reelin	[25]
	2015	DAB1	[26]
	2016	ApoER2	[27]
Rescue by overexpression in disease model animals	2014	reelin	novel object recognition rescued in the AD model mice strain J20 [28]
	2020	reelin	passive avoidance rescued in τ-overexpressing mice [29]
Processes related to hippocampal neurogenesis supported by molecules of the reelin signaling pathway	2004	reelin	dendritogenesis [30]
	2008	reelin	dendritic spine development [31]
	2012	DAB1	neurogenesis [32]
	2016	reelin	synaptogenesis [33]
